# Prevalence of and risk factors for postoperative delirium among children after cardiac surgery in a Single-Centre retrospective study

**DOI:** 10.1038/s41598-025-04927-z

**Published:** 2025-06-20

**Authors:** Sophia Schumann, Gerhard Schön, Ida Hüners, Daniel Biermann, Lena Christine Siebel, Friederike Jess, Urda Gottschalk, Carolin Gleitze-Nolting, Jonas Denecke, Johannes Drescher, Dominique Singer, Michael Hübler, Rainer Gerhard Kozlik-Feldmann, Sebastian Hermann Harms

**Affiliations:** 1 Department of Paediatric Cardiology, Children’s Heart Clinic, University Heart & Vascular Centre, University Medical Centre Hamburg-Eppendorf, Martinistr. 52, 20246 Hamburg, Germany; 2 Institute of Medical Biometry and Epidemiology, University Medical Centre Hamburg-Eppendorf, Hamburg, Germany; 3 Department of Congenital and Paediatric Heart Surgery, Children’s Heart Clinic, University Heart & Vascular Centre, University Medical Centre Hamburg-Eppendorf, Hamburg, Germany; 4 Department of Paediatrics, Centre for Obstetrics and Paediatrics, University Medical Centre Hamburg-Eppendorf, Hamburg, Germany; 5 Department of Child and Adolescent Psychiatry and Psychotherapy, Centre for Psychosocial Medicine, University Medical Centre Hamburg-Eppendorf, Hamburg, Germany

**Keywords:** Paediatric delirium, Cornell assessment of pediatric delirium (CAPD), Neurodevelopment, Congenital heart disease (CHD), Iatrogenic withdrawal syndrome (IWS), Paediatric cardiac intensive care unit (pCICU), Paediatric research, Congenital heart defects, Disorders of consciousness

## Abstract

Due to the increasing focus on neurodevelopment in children with congenital heart disease (CHD), early predictive markers are crucial for implementing interventions and improving neurodevelopmental outcomes. As postoperative delirium (PD) is known to have a long-term impact on neurocognitive function in adults, studies on the prevalence of and modifiable risk factors for PD offer new perspectives. We conducted a retrospective, single-centre study screening for PD using the Cornell Assessment of Pediatric Delirium (CAPD). We distinguished PD from iatrogenic withdrawal syndrome (IWS) by using the Withdrawal Assessment Tool 1 (WAT-1). A confirmatory, multivariate regression analysis was performed and included various pre-, intra-, and postoperative variables. The screening compliance rate was 95% among the 311 patients. The prevalence of PD was 40.2%, and 46.4% of the patients developed IWS. Infants were at the highest risk for PD (OR 2.9, *p* = 0.05). Prolonged mechanical ventilation > 100 h (OR 7.4, *p* = 0.003), infusion therapy with ketamine (OR 3.3, *p* = 0.009), IWS (mild: OR 7.7, p = < 0.001, severe: OR 17.0, p = < 0.001) and low cardiac output syndrome (LCOS) (OR 3.9, *p* = 0.02) were significant predictive risk factors for PD. Overall, PD and IWS are highly prevalent in paediatric cardiac intensive care units (pCICUs), especially in infants and children with prolonged ventilation durations who require multiple sedatives. This is one of the most extensive single-centre studies in the pCICU population, and the results revealed that IWS and lactatemia in the context of LCOS are novel predictors of PD.

## Introduction

Congenital heart disease (CHD) remains the most common form of congenital disability worldwide, with a prevalence of 18 per 1000 live births; furthermore, CHD accounts for 4% of neonatal deaths^1^. Fortunately, the mortality of CHD has significantly decreased in recent decades^2^. Therefore, investigations on morbidity and long-term outcomes such as neurodevelopment are needed.

While one-third of children who undergo cardiac surgery for CHD suffer from developmental delays that involve either one or combined areas of motor, language, or cognitive skills^3^the degree of impairment is linked to the severity of CHD^4^. Furthermore, each postoperative day a child spends in the paediatric cardiac intensive care unit (pCICU) equates to losing one point in the intelligence quotient^5^. Moreover, the pCICU length of stay is negatively correlated with the development of motor skills^6^.

In addition to neurodevelopmental outcomes, post-cardiac surgery psychological disorders in children, such as anxiety, posttraumatic disorders, or depression, are frequent and affect the quality of life (QoL) of patients as well as their families to a variable degree^7,8^.

While two-thirds of CHD patients do not suffer from neurological impairment, it is important to determine methods for identifying patients who will have neurodevelopmental delays later as early as possible in order to provide appropriate support to patients and families.

While there are established assessment tools for infants and toddlers, such as the Bayley Scales of Infant Development, the links between these assessment tools and early predictors of developmental delay remain unclear, as the use of postoperative neuromonitoring as a possible predictive tool is very heterogeneous, according to an extensive European survey^9^. In some studies, pre- and postoperative neuroimaging is used to identify complications such as stroke, white matter injury, or a reduction in brain volume in CHD patients; furthermore, these imaging findings have been shown to be correlated with neurodevelopment^10–12^. Biomarkers indicating neurological damage, such as glial fibrillary acidic protein, which increases during and after bypass surgery in children^13^seem to be promising components for predicting neurodevelopment^14^.

Few studies on neurodevelopment have examined data from the perioperative course of children undergoing cardiac surgery. While technical applications of neuromonitoring, such as near-infrared spectroscopy (NIRS) and amplitude-integrated electroencephalography (aEEG), are increasingly frequently used in pCICUs and are correlated with neurodevelopmental outcomes^15,16^simply assessing the postoperative clinical presentation of neuropsychological dysfunction might be another key component. In particular, these scoring tools are comparably cost-effective and do not need require neuroimaging or laboratory testing. Screening for paediatric delirium (PD), even in neonates and infants, who account for the vast majority of pCICU patients, has recently become possible with the development of the Cornell Assessment of Pediatric Delirium (CAPD)^17^. The CAPD has shown excellent concordance with the Diagnostic and Statistical Manual of Mental Disorders 5 (DSM-5) criteria^18^ and has been validated for the German language^19^. International guidelines recommend its use^20,21^. Nevertheless, in an extensive survey, only 44% of centres have implemented regular delirium screening in paediatric ICUs (PICUs)^22^.

Delirium is defined as a neuropsychiatric disorder and is associated with disturbances in consciousness and attention, alterations in perception, disorganised thinking, acute onset, and a fluctuating course. The pathophysiology of delirium has not yet been fully elucidated. Hypotheses regarding its pathophysiology are based on an imbalance of neurotransmitters in the central nervous system (CNS) and dysfunction of neuronal networks. Neuroinflammatory components are suspected to contribute to delirium^23,24^whereas proinflammatory cytokines lead to increased permeability of the blood‒brain barrier (BBB)^25^thus increasing the vulnerability of the CNS and resulting in the so-called “failure of the system integration hypothesis”^26^.

Various risk factors for delirium have been described in the elderly population, including infection, drug withdrawal, endocrine disorders, trauma, CNS pathology, hypoxia, vitamin deficiencies, acute vascular disorders, medications, and heavy metal exposure^27^. Many of these risk factors apply to CHD patients, particularly their perioperative course, thus indicating a high-risk profile for developing delirium in this cohort.

Postoperative delirium in adults is generally associated with an unfavourable outcome and occurs with a high prevalence in the intensive care unit, especially after major or cardiac surgery^28,29^. Meta-analyses have demonstrated that postoperative delirium is associated with neurological impairment as well as psychological disorders^28,30–32^.

Since the development of CAPD, studies have evaluated the prevalence of PD in paediatric intensive care settings. A one-day, multipoint-prevalence study of twenty-seven pCICUs and PICUs revealed a prevalence of 40%^33^. These results are consistent with the findings of a recent review^34^. The highest risk occurs in paediatric patients treated with extracorporeal membrane oxygenation (ECMO)^35^. Nevertheless, the prevalence is highly variable between different monocentric studies, ranging from 25% to almost 68% ^36–39^, most likely due to different treatment variables between the centres, indicating the possibility of yet unknown modifiable risk factors.

Up to date only patient age and duration of mechanical ventilation have been identified as definite risk factors for PD. Moreover, developmental delay, cyanotic heart disease, cardiopulmonary bypass time (CPBT), and elevated pain scores are likely to be associated with PD^34^.

The high prevalence of delirium in the postoperative paediatric population and long-term results in the adult population imply that PD might cause developmental delay in CHD patients or at least be a surrogate parameter for perioperative neurological damage and may act as an early predictor of impaired neurodevelopment.

## Methods

### Aims, outcomes, and study design

We conducted a retrospective single-centre observational cohort study over 15 months from March 2023 to June 2024 to evaluate the prevalence of risk factors for PD in our cohort by conducting a confirmatory analysis.

The primary endpoint was as follows:

- Prevalence of postoperative delirium after cardiac surgery with or without cardiopulmonary bypass in all children aged 0–18 years.

Perioperative risk factors related to postoperative delirium, including possible factors in the paediatric and elderly populations, were identified by conducting an extensive literature review.

The secondary endpoints included the following variables:

- Patient age at the time of surgery, divided into four groups: (1) neonates (0–1 month), (2) infants (1–12 months), (3) toddlers (1–6 years), and (4) schoolchildren/adolescents (7–18 years).
- Risk stratification of the type of surgery according to the Risk Adjustment for Congenital Heart Surgery (RACHS-1) score^40^.
- Univentricular physiology.
- Cardiopulmonary bypass time.
- Hypoxia (transcutaneous saturation (SaO2) of < 92% for more than six consecutive hours).
- Metabolic derailment (blood glucose level > 200 mg/dl in blood gas analysis (BGA)).
- Lactatemia (> 2 mmol/l over six consecutive hours in arterial BGAs).
- Postoperative fever (> 38.5 °C for more than three consecutive hours).
- Signs of postoperative infection (postoperative fever and secondary increase in C-reactive protein (CRP) not due to second surgery and/or pathological microbiological findings in blood, tracheal or urinary samples and/or restart of antibiotic treatment due to clinical presentation of infection).
- Postoperative use of hydrocortisone to enhance inotropic effects and stabilise haemodynamics due to signs of systemic inflammatory response syndrome (SIRS).
- Time on ventilation (divided into four groups): (1) on-table extubation (OTE), (2) fast-track (0–10 h (h), (3) short-term ventilation (> 10–100 h), and (4) long-term ventilation.

For subgroup analysis, patient data concerning the following conditions were obtained for patients who remained ventilated in the pCICU (ventilation groups 2–4):

- COMFORT-B scale.
- The use of three different sedative infusions: (1) dexmedetomidine, (2) ketamine, and (3) midazolam.

To differentiate between lactatemia type A (resulting from inadequate delivery of oxygen and tissue hypoxia) and type B (resulting from metabolic uncoupling after bypass)^41,42^the following data were extracted for subgroup analysis:

- Arterio-venous difference in O_2_ concentration (avDO2) > 40%.
- Differences in central to peripheral temperature (ΔT) > 4 °C.

All secondary endpoints were registered before the first CAPD score was evaluated. The exclusion criteria included ICU admission for minor procedures such as central venous catheter (CVC) or pleural drain placement with short-acting sedation or admission for noncardiac surgery conditions. The study adhered to the Helsinki Declaration, and owing to the retrospective nature of the study, the Hamburg Medical Association Ethics Committee waived the need to obtain informed consent (waiver 2023-300407-WF).

## Sample size calculation

The primary goal of the study was to estimate the prevalence of PD using the CAPD screening tool. A 95% confidence interval not greater than ± 6% was considered clinically relevant. We assumed the prevalence to be between 40% and 50%. Therefore, a 40% prevalence would require *n* = 271 cases, and a 50% prevalence would require *n* = 281 cases. We planned conservatively and decided on a sample size of *n* = 281 cases. To account for an assumed dropout of 2%, *n* = 287 cases were included in the study.

## Patient characteristics

The authors’ pCICU is located within a large tertiary centre with a variety of treated cardiac malformations, ranging from simple to complex univentricular heart defects, with the possibility for veno-arterial (VA)- and veno-venous (VV)-ECMO treatment when needed. Since the introduction of fast-track protocols^43^ in our centre in 2018, more than half of all patients have either been extubated in the theatre or on the same day of surgery in the pCICU. Nearly all postoperative patients are first-line sedated with dexmedetomidine (0.5–1.4 µg/kg/h), whereas additional continuous sedation with ketamine or midazolam is avoided whenever possible according to guideline recommendations. Muscle relaxation (rocuronium 0.6–1.0 mg/kg/h) is only used in high ventilation settings to prevent barotrauma. The first-line analgesic treatment in our centre is either single-dose opioids (morphine or piritramide 0.1 mg/kg/dose) when the patient is extubated or remifentanil infusion (0.05–2.0 µg/kg/min) when short-term ventilation is expected. In challenging treatment courses, owing to increasing tolerance, drug cycling to morphine (30–100 µg/kg/h) or sufentanil (0.3–1.3 µg/kg/h) perfusion becomes necessary. Weaning protocols after prolonged sedation and opioid exposure are standardised and guided by scoring for patient comfort (COMFORT behaviour (COMFORT-B)) and withdrawal (Withdrawal Assessment Tool 1 (WAT-1)).Supplementary Figure 1 provides further details about our decision and treatment algorithm.  

## Patient assessment for pain, iatrogenic withdrawal syndrome, and postoperative delirium

PD was screened using the CAPD and differentiated from symptoms of iatrogenic withdrawal syndrome (IWS) using the WAT-1 ^44^ only when patients were awake, as classified by a Richmond agitation and sedation score (RASS)^45^ of ≥ -2. Comfort and pain were assessed using the COMFORT-B scale. All scores were obtained every eight hours by pCICU nurses and doctors.

The CAPD is structured around the DSM-5 criteria and includes eight questions scored from 0 to 4 points, yielding a total score of 0 to 32 points, with delirium indicated by a score of ≥ 9. To address staff variability in the recognition of delirium in pCICU patients, “development anchor points” were established for children up to 2 years of age, outlining normal versus atypical behaviours by age^19^. The WAT-1 score includes 11 questions, which are scored from 0 to 2 points, with a total score of 12. We divided the score into ≥ 3 points, mild, and ≥ 7 points as severe IWS. The COMFORT-B comprises six questions, scoring from 0 to 5 points, with a total score of 30. A score between 11 and 22 points is considered adequate sedation and pain treatment, whereas a score < 11 points indicates an oversedated patient, and a score > 22 points indicates an undersedated patient^46^.

## Data collection

Data were extracted from the electronic medical record (EMR) *Integrated Care Management* (ICM, version 13.02 by Drägerwerk AG&Co. KGaA was performed using ICMiq software (version 1.5.0) by Drägerwerk AG&Co. KGaA. Before analysis, a trustee pseudonymized all records provided by the Data Integration Centre.

### Statistical analysis

Throughout this study, we used parametric statistics measures, such as arithmetic means and standard deviations, and categorical variables, frequencies, and proportions. The p values were obtained using t tests or chi square tests. For multivariate modelling, we calculated logistic regression models and reported the odds ratios, confidence intervals of the odds ratios, and p values. All calculations and figures were made using the statistical program R (version 4.4.1, by the R Foundation for Statistical Computing, Vienna, Austria)^47^.

## Results

**Fig. 1:**
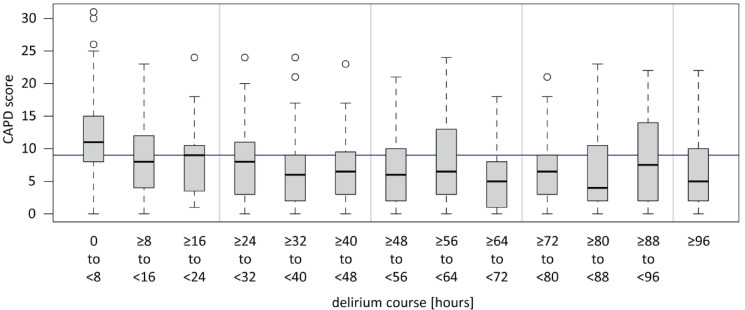
Duration of delirium in all delirious patients (*n* = 118). Patients were screened positive for delirium when the CPAD score was ≥ 9 points for the first time (time of delirium hour 0). CAPD was performed as soon as the patient was awake (RASS ≥-2). CAPD: Cornell Assessment of Pediatric Delirium, RASS: Richmond Agitation and Sedation Scale.

From March 2023 to June 2024, a total of 387 patients were treated for surgical and medical conditions in the pCICU. After excluding patients who were treated for medical conditions and short-term procedures, 311 patients treated after heart surgery with or without CPB remained. With a compliance rate of 94.2%, the CAPD and WAT-1 were administered to 293 of these 311 patients (Table 1). Delirium occurred in 118 (40.2%) patients, and IWS occurred in 136 (46.4%) patients, including 109 patients with mild IWS symptoms and 27 with severe IWS symptoms. The average duration of delirium was 53 h, with most patients presenting with delirium directly after extubation but also later, partially with a fluctuating course, as demonstrated in the box plot in Fig. 1.

**Table 1:**
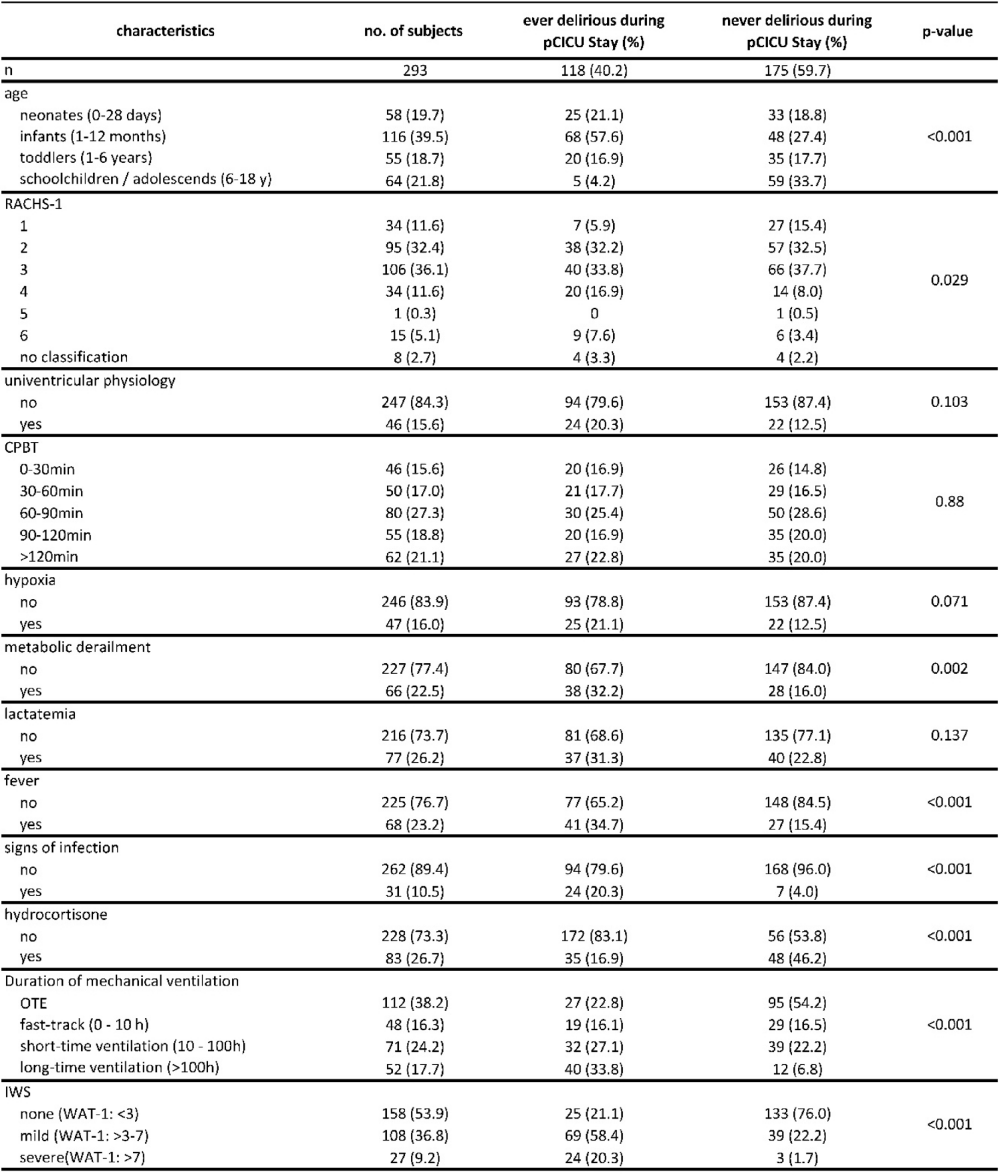
Demographic and clinical characteristics by delirium status (*n* = 293), RACHS-1: risk adjustment for congenital heart surgery, CPBT: cardiopulmonary bypass time, IWS: iatrogenic withdrawal syndrome, WAT-1: withdrawal assessment tool 1, OTE: On-table extubation.

**Fig. 2:**
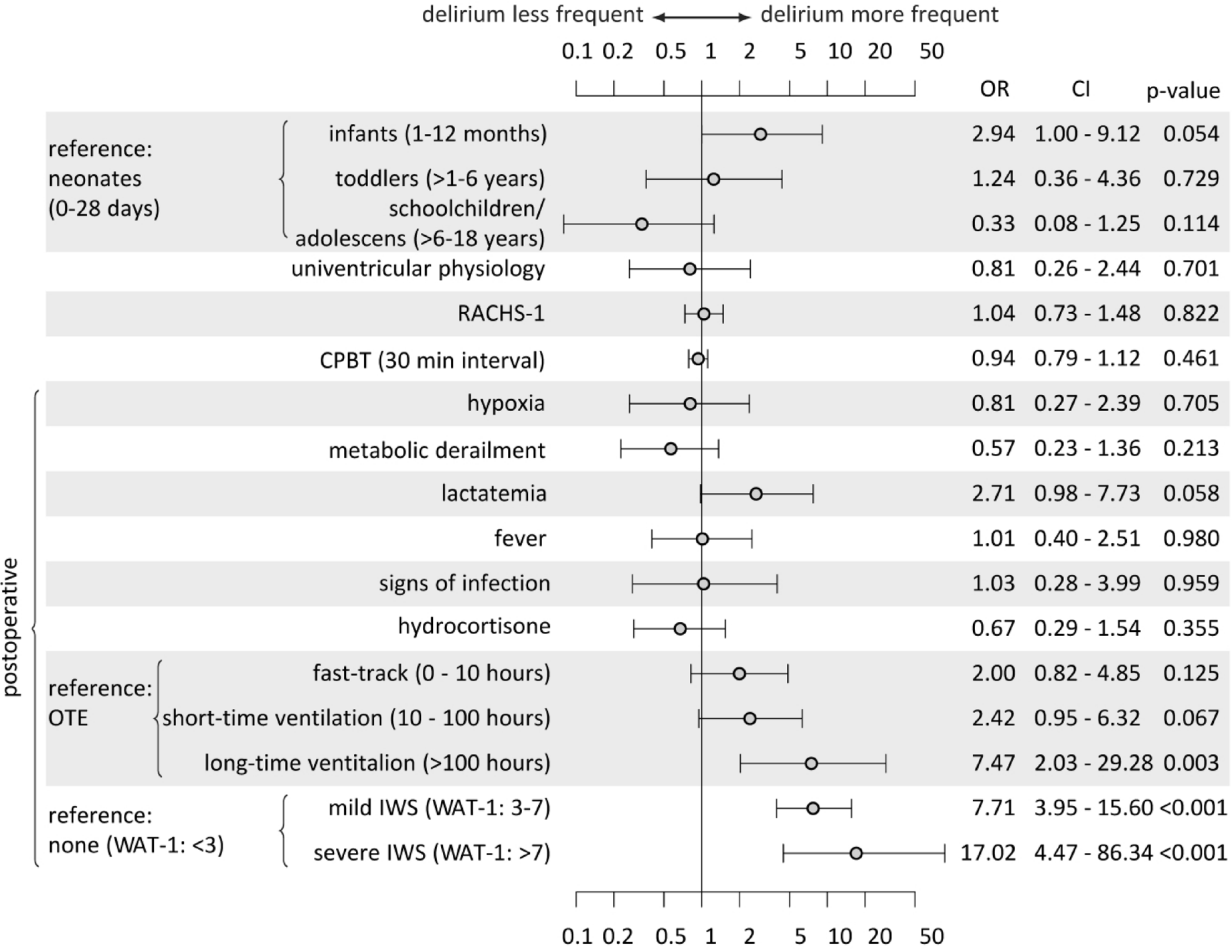
Multivariate logistic regression model for predicting postoperative delirium in children after cardiac surgery with or without bypass (*n* = 293). OR: Odds Ratio, CI: Confidence interval, WAT-1: Withdrawal Assessment Tool 1, IWS: Iatrogenic withdrawal syndrome, OTE: On-table extubation, RACHS-1: Risk Adjustment for Congenital Heart Surgery, CPBT: Cardiopulmonary bypass time.

Multivariate regression analysis was conducted using data from 293 patients (Fig. 2). After controlling for age, school-aged children and adolescents had the lowest delirium rates (OR 0.3, *p* = 0.1), and infants had the highest delirium rates (OR 2.9, *p* = 0.05) compared with neonates. The RACHS-1 (OR 1.0, *p* = 0.8) and CPBT (OR 0.9, *p* = 0.4) were not correlated with delirium, with a narrow confidence interval and a precise effect size.

The presence of IWS was a robust predictor of PD, categorised as mild IWS (OR 7.7, *p* < 0.001) and severe IWS (OR 17.0, *p* < 0.001). Compared with those of OTE, delirium rates increased with increasing length of ventilation, particularly for long-term ventilated children (OR 7.4, *p* = 0.003). Patients who had elevated lactate levels for more than six consecutive hours had substantially higher delirium rates (OR 2.7, *p* = 0.05). No trend was identified for hypoxic or univentricular patients. Metabolic derailment, postoperative fever, signs of infection, and postoperative use of hydrocortisone did not predict delirium.

**Fig. 3:**
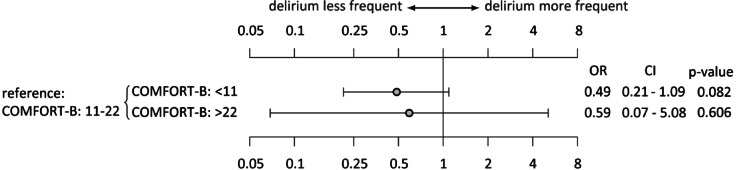
COMFORT-B scores for ventilated patients who were transferred to the pCICU (*n* = 154). The reference COMFORT-B score of 11–22 points indicates an adequate level of sedation while the patient is intubated. The patient was considered to be oversedated if the score was less than 11 points, with more than 22 points undersedated. OR: odds ratio, CI: Confidence interval.

A total of 187 patients remained ventilated after being transferred to the pCICU. COMFORT-B scores were obtained every shift (8 h). Because the fast-track protocol was carried out for some of these patients before scoring, only 154 patients had a COMFORT-B score before extubation. As demonstrated in Fig. 3, all patients who remained ventilated with a low COMFORT-B score < 11 had a lower delirium rate after extubation (OR 0.4, *p* = 0.08) than patients with a COMFORT-B score 11–22.

**Fig. 4:**
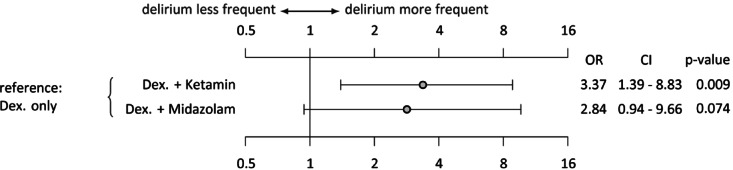
Different continuous infusion therapies predict delirium in children who remain ventilated after cardiac surgery (*n* = 146). Dex: Dexmedetomidine, OR: Odds ratio, CI: Confidence interval.

Nearly all postoperative patients received dexmedetomidine infusion in our centre, even if they were extubated in the operating room, to modulate pain perception and reduce the need for opioids. If the patient required additional sedation to achieve an adequate sedation level (COMFORT-B 11–22) and remained tube-tolerant, ketamine or midazolam was added as infusion therapy if single bolus therapy was not sufficient. Figure 4 shows that escalating to ketamine infusion therapy significantly increased delirium rates (OR 3.3, *p* = 0.009). This is also the case for escalation to midazolam infusion therapy, although the increase was not significant (OR 2.84, *p* = 0.07).

**Fig. 5:**
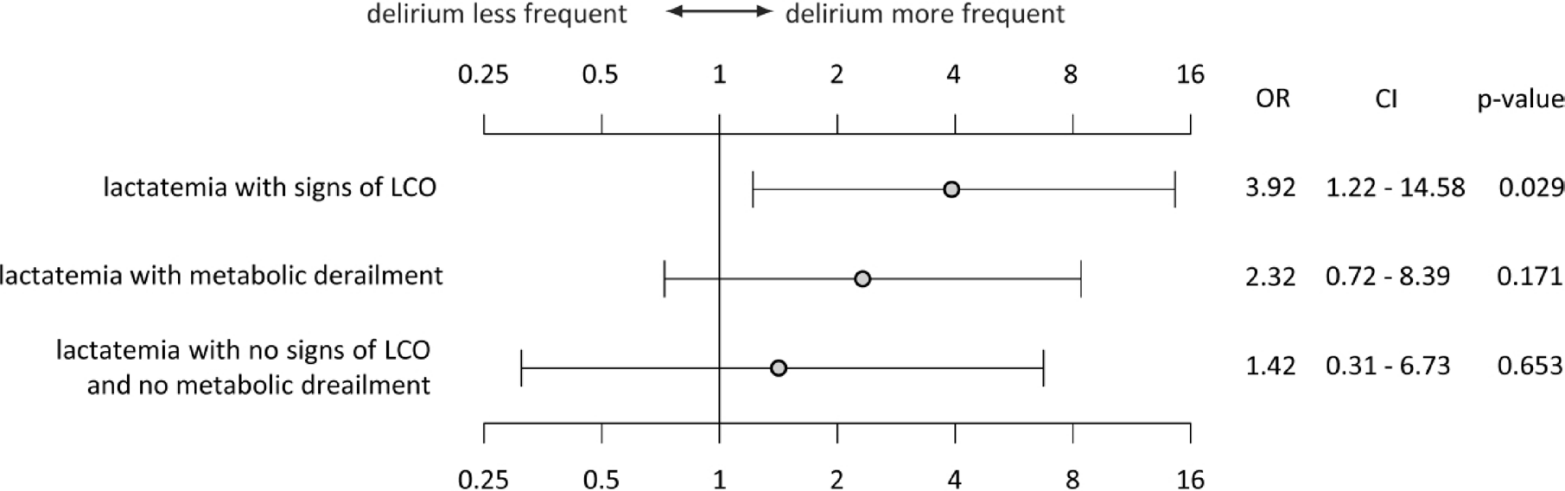
Subgroup analysis of patients with different causalities for postoperative lactatemia and delirium (*n* = 83) OR: Odds ratio, CI: Confidence interval.

Owing to the substantial correlation between postoperative lactatemia and delirium (Fig. 2), we conducted a subgroup analysis of all 83 patients with consecutive lactatemia over more than six hours (Fig. 5) to distinguish whether the elevated lactate was attributable to low cardiac output (LCO) (lactate acidosis type A) or to metabolic derailment (lactate acidosis type B).

Patients with signs of low cardiac output (*n* = 19), defined as lactatemia for more than 6 h, AVDO2 > 40%, and a core-to-peripheral temperature gradient of > 4 °C, had a significantly higher delirium rate (OR 3.9, *p* = 0.02) than patients with metabolic uncoupling, defined as lactatemia and a blood glucose level > 250 mg/l.

## Discussion

With a CAPD implementation rate of approximately 95%, we observed a 40.2% prevalence of delirium. This is consistent with the pooled prevalence of 39% in pCICUs observed in a review^34^ and the prevalence of 40% reported in the only multicentre study to date^33^. According to a systematic review by Ista et al., the pooled prevalence in pCICUs, 53% (four studies) is higher than that in PICUs (23%, 17 studies)^48^.

### Patient’s age and duration of mechanical ventilation predicts delirium

In our study, infants had the highest risk of developing PD (OR 2.9, *p* = 0.05), and schoolchildren/adolescents had the lowest risk (OR 0.3, *p* = 0.11). This finding is consistent with those of previous studies that reported that children younger than 2 years^37^ or younger than 1 year^36^ have a significantly greater risk of developing PD. Our study confirms that mechanical ventilation duration is associated with increased rates of PD and a significantly increased risk in those with more than 100 h of ventilation (OR 7.3, *p* = 0.003). These results are consistent with the current literature^33,37,49^ and most likely reflect the cumulative effects of various variables contributing to a longer ventilation time. Notably, compared with patients with OTE, even patients on fast-track protocols (0–10 h, OR 2.0, *p* = 0.12) or ventilated for 10–100 h (OR 2.4, *p* = 0.06) had a substantially greater risk of developing PD. This might reflect the benefits of the differences in pain and sedation management by anaesthetists in theatres compared with management in the pCICU, and further evaluation is needed. Overall, this highlights the importance of OTE, which, in our experience, is not pursued in all possible cases due to logistical issues in theatres.

### Severity of cardiac surgery and time of cardiopulmonary bypass do not predict delirium in our cohort

In contrast with other studies, RACHS-1 did not predict PD in our cohort. While Patel et al. reported a significant difference in the prevalence of delirium between RACHS-1 groups 1 and 2 and RACHS-1 groups 3 and 4 ^37^, our study revealed no correlation (OR 1.0 *p* = 0.82, CI of 0.73 to 1.48). Köditz et al. also did not find a correlation between the RACHS-1 and CAPD, but the vast majority of patients had a RACHS-1 score of either 2 or 3 ^50^, so the comparison might not be feasible. Alvarez et al. did not use the RACHS-1 score to assess surgical complexity in their study; however, the alternative Society of Thoracic Surgeons and European Association for Cardio-Thoracic Surgery (STS-EACTS) scale was significantly correlated with delirium^36^. Mao et al., on the other hand, focused on the general severity of illness and used the PRISM (Pediatric Risk of Mortality) III score, which was also significantly associated with delirium^49^.

Compared with the findings of Alvarez et al.^36^the CPBT per 30-minute interval did not predict delirium in our cohort (OR 0.94, CI 0.79–1.12, *p* = 0.46). The multicentre study by Staveski et al.^33^ and the final multivariate analysis by Mao et al.^49^ also did not demonstrate a significant correlation.

Further research is needed to analyse the details of perfusion and surgical techniques regarding the prevalence of delirium. The inconsistency in the current literature may indicate further, possibly modifiable risk factors.

### Sedation strategy and patient comfort impact delirium, while the presence of iatrogenic withdrawal syndrome is the strongest predictor of delirium

A COMFORT-B score of less than 11, which usually represents oversedation^46^was a protective factor against delirium (OR 0.49, p 0.08). However, the COMFORT-B score comprises several aspects. On the one hand, it assesses pain, which has been identified as a possible contributor to delirium^51^. Consequently, children with highly potent analgesia for short-term sedation have a significantly lower rate of delirium^52^. On the other hand, oversedation, which is measured by EEG during anaesthesia for paediatric cardiac surgery, was shown to promote delirium^50^. Continuous EEG monitoring was not implemented in our pCICU during the study period. Therefore, no further information can be provided. Assuming that a COMFORT-B score < 11 points does not necessarily equate to a complete suppression pattern in EEG monitoring, there may be optimal sedation and analgetic depth to reduce delirium rates that are below the standard range of the COMFORT-B score of 11–22 points but still far from a postoperative EEG suppression pattern. Further research is needed to evaluate this relationship. Notably, CAPD scoring was only performed during the pCICU stay. Therefore, owing to individual pharmacokinetics, patients with a COMFORT-B score < 11 during intubation might have developed delirium after transfer from the pCICU, as fluctuating and late-onset delirium courses were observed during the pCICU stay (Fig. 1).

Sedation and analgesia adjustments were made by the attending intensivist and nursing staff based on COMFORT-B score and in accordance with our standard operating procedure (Supplementary Fig. 1) and international guidelines^20^. We used a standardised protocol of single-dose opioid and dexmedetomidine infusion and used only ketamine and midazolam infusions as second- and third-line treatments. Nevertheless, long-term ventilation and increased drug tolerance sometimes require escalation. Benzodiazepine exposure is a known risk factor for delirium in the adult population^53^ and in pCICU patients^36^. The subgroup analysis for ventilated patients revealed a favourable delirium rate if dexmedetomidine was the only sedative used compared with a combination of ketamine (OR 3,3, *p* = 0.009) or midazolam infusion (OR 2.84, *p* = 0.07). These findings are conclusive with data from postoperative children with congenital heart disease and pulmonary hypertension (PAH)^54^as well as in large randomised trials in the adult population^55^.

IWS can be misinterpreted as delirium without scoring tools, although the two syndromes significantly differ in terms of aetiology and symptoms. Current international guidelines suggest that clinical evaluations should include IWS and delirium scoring^20^. Our study also emphasises this difference. In our research, IWS was identified with high statistical significance (mild IWS OR 7.7, *p* < 0.001 and severe IWS OR 17.0, *p* < 0.001) for the first time in a large monocentric pCICU population as a predictive factor for PD. To date, these results have only been described in the multicentre 1-day study by Staveski et al. In this study, WAT-1 levels were significantly higher at the time of delirium diagnosis, and the patient had a higher WAT-1 score twenty-four hours before being diagnosed with delirium^33^. In contrast, we chose a confirmatory study design, which minimises type I error (alpha error) that can occur in exploratory model building. A methodological limitation lies in the overlap of the diagnostic question “Is the child restless?” within the two scoring questionnaires, WAT-1 and CAPD. This question, which occurs in both scores, could lead to diagnostic overlap and, thus, a potential distortion of the differential diagnosis between IWS and delirium. However, since withdrawal is a well-known risk factor in the elderly population^27^its predictive value remains evident from our point of view.

These results might lead to new preventive approaches. Using more adequate analgesics during ventilation to reduce pain and optimising weaning protocols to prevent IWS might be key components in preventing PD, as the amount of opioid exposure predicts IWS^56^. A large prospective multicentre trial recently demonstrated that intermittent intravenous paracetamol-based analgesia can substantially reduce opioid use^57^.

Overall, further evaluation of multimodal, opioid-sparing analgesia protocols is warranted, and minimising the use of midazolam or ketamine should play an essential role in PD prevention. EEG monitoring for sedation depth in ventilated pCICU patients needs to be investigated, and it might prevent oversedation. Further development of fast-track protocols for anaesthesia and intensive care could reduce delirium rates in children after cardiac surgery.

### Postoperative low cardiac output but not hypoxia predicts delirium

In the multivariate analysis, we identified postoperative lactatemia as a novel significant risk factor for PD in the pCICU cohort (OR 2.7, *p* = 0.05). This variable was not investigated in previous pCICU studies, and data in the adult population support our findings by demonstrating a significant correlation between lactatemia and postoperative delirium^58^. We further differentiated the origins of lactatemia^59^ using additional signs of low cardiac output syndrome (LCOS), such as AvDO2 > 40% and a core-to-peripheral temperature difference > 4 °C, to circumscribe the LCOS definition. To our knowledge, this is the first study to identify hyperlactatemia type A (LCOS) but not metabolic uncoupling (hyperlactatemia type B) as a significant predictor of postoperative delirium after cardiac surgery (OR 3.9, *p* = 0.02). Mao et al. reported that secondary thoracic closure was predictive of delirium^49^. Oftentimes, delayed sternal closure is due to a certain state of LCOS, which indicates a possible correlation. On the one hand, prolonged hypoxia or univentricular physiology were not significant predictors of PD according to our multivariate analysis. Liu et al. demonstrated a preventive effect for delirium by optimising regional cerebral oxygen saturation^60^which would, on the other hand, support our novel findings concerning the LCOS. Real-time NIRS monitoring is available for all our patients. Nevertheless, owing to its technical compatibility, it is not documented in sufficient detail in the EMR; thus, this effect cannot be demonstrated in our analysis.

### Fever, infection, and use of steroids do not seem to predict delirium

In contrast to the findings of a previous study by Meyburg et al.^61^postoperative fever and infections were not significant risk factors for PD in our study. However, antipyretic pain medications (paracetamol and metamizole) are administered per the standard protocol, every six hours for every patient in our cohort. Active temperature control systems are used for patients with signs of LCOS, and in one-third of all patients, postoperative hydrocortisone is administered. Therefore, our data are likely not consistent enough to answer the question of how the immune system influences the occurrence of postoperative delirium. The role of neuroinflammation has generally been described in the literature^23,24^. Further research is needed to better characterise the influence of neuroinflammatory processes on the development of delirium in the context of postoperative care in this specific patient population.

### Study strengths and limitations

With 311 patients and a compliance of nearly 95%, we obtained accurate prevalence rates in our cohort and therefore performed a detailed risk factor analysis. Compared with previous studies, an evaluation every 8 h is more precise in detecting fluctuating forms of delirium. Nevertheless, there are several limitations. As a single-centre observational cohort study, our data cannot be widely generalised, and no causality can be drawn. We did not determine the delirium subtypes (hypo-, hyperactive, or mixed). CAPD and WAT-1 scoring did not continue after transfer from the pCICU; therefore, some cases may have been missed due to late appearance. Additionally, children with a positive delirium score were treated immediately for ethical reasons (environmental modifications and, in rare cases, the use of antipsychotic medication), which could have influenced the course and duration of delirium. While several predictive risk factors were identified in our study, there are undoubtedly other, yet unknown, postoperative and intraoperative risk factors that were not recorded in our analysis and may play a role in delirium. These limitations emphasise the need for multicentre studies using larger databases and prospective interventional studies to identify preventive strategies.

## Conclusion

PD and IWS are highly prevalent after paediatric cardiac surgery, and screening should be part of standard care. While nonmodifiable risk factors such as patient age play an important role in risk stratification, modifiable risk factors such as the duration of mechanical ventilation emphasise the further development of fast-track protocols. Since we described LCOS as a novel risk factor, neuromonitoring should aim to optimise cerebral tissue perfusion. As we demonstrated, IWS is a strong predictor of delirium, and PD prevention demands not only well-considered sedation and adequate opioid-sparing pain management and avoidance of ketamine and benzodiazepine use but also sophisticated weaning protocols. Overall, further research is needed to determine the predictive value of postoperative delirium in neurodevelopment, as infants have the highest prevalence, and data in the adult population are predictive.

## Electronic supplementary material

Below is the link to the electronic supplementary material.

Supplementary Material 1

## Data Availability

The dataset used and/or analysed during the present study is available from the corresponding author upon reasonable request.
