# Supplementary material for: Prevalence of and risk factors for postoperative delirium among children after cardiac surgery in a Single-Centre retrospective study
Source: Sci Rep. 2025 Jun 20;15:20140. doi: 10.1038/s41598-025-04927-z (PMC12181358; doi:10.1038/s41598-025-04927-z)
Supplement: Supplementary file 1 — Supplementary Material 1 [file 41598_2025_4927_MOESM1_ESM.docx]

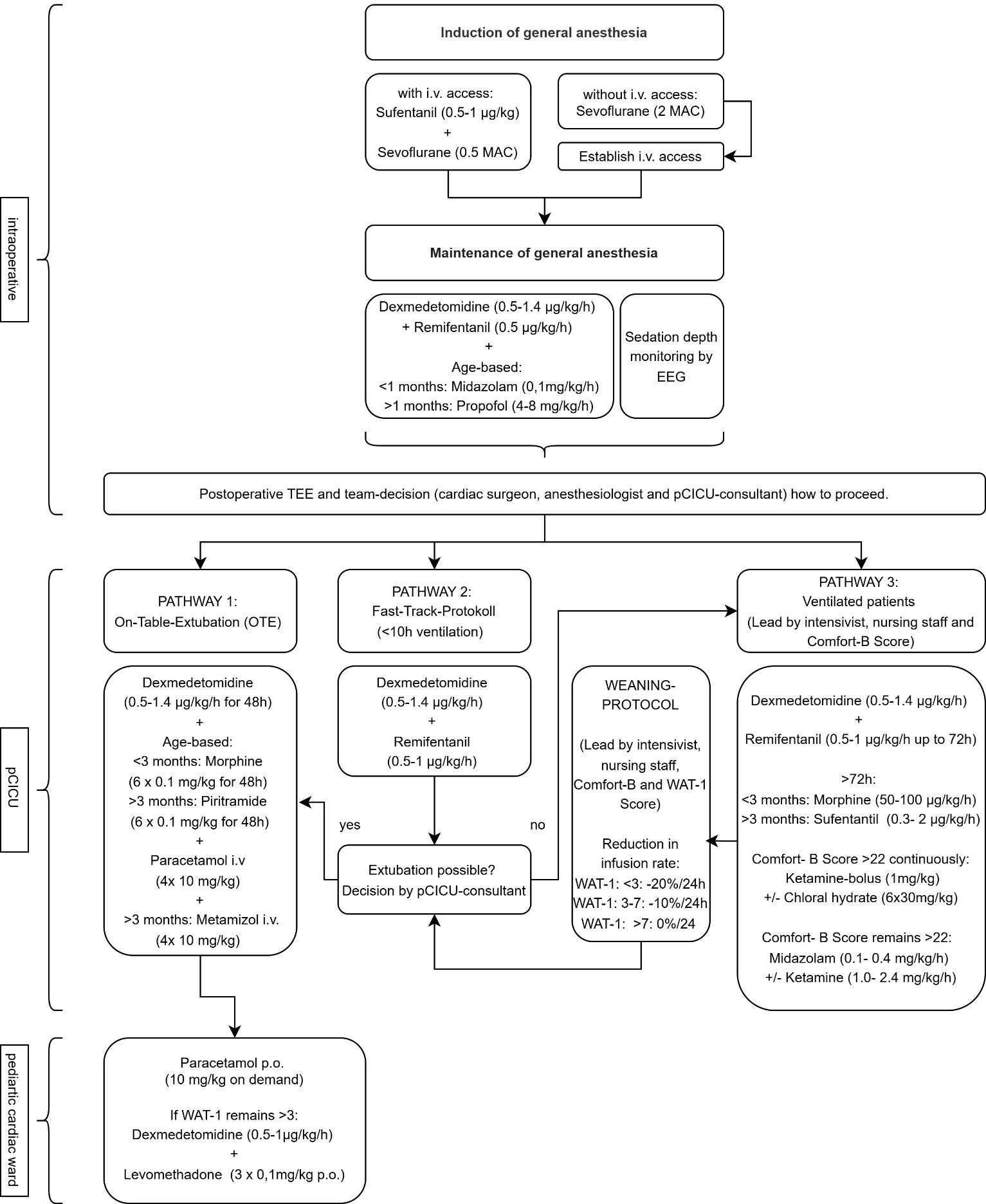


**Supplementary Figure 1:** Algorithm for Perioperative Analgosedation. I.V. Intravenous, MAC: Mean airway concentration, EEG: Electroencephalogram, TEE: Transoesophageal echocardiography, OTE: On-table-extubation, pCICU: Paediatric cardiac intensive care unit, WAT-1: Withdrawal Assessment Tool 1, p.o.: Per os
